# A Mean and Miserable Diet

**Published:** 1890-07-26

**Authors:** 


### The Hospital

A Weekly Journal of

**Science, Medicine, Nursing, and Philanthropy.**

*For Hospitals, Asylums, Medical Practitioners, Students, Nurses, Families, Parishes, Congregations, and the Charitable Public.*

Vol. VIII.—No. 200.] [July 26, 1890.

### A Mean and Miserable Diet.

The vegetarians will be put on their mettle by a passage which we will quote for their and the general benefit from Mr. Stanley's last and greatest book. Says the traveller: "I was never so sensible of the evils of forest marching as on this day. My own condition of body was so reduced, owing to the mean and miserable diet of vegetables on which I was forced to subsist, that I was more than usually sympathetic. At this time there were about thirty naked Madis in the last stages of life; their former ebony black was changed to an ashy-grey hue, and all their bones stood out so fearfully prominent as to create a feeling of wonder how such skeletons were animated with the power of locomotion. Almost every individual among them was the victim of some hideous disease; and tumours, scorched backs, fœtid ulcers, were common; while others were afflicted with chronic dysentery, and a wretched debility caused by insufficient food. A mere glance at them, with the malodour generated by their ailments, caused me to gasp from a spasm of stomach sickness. With all this, the ground was rank with vegetable corruption, the atmosphere heated, stifling, dark, and pregnant with the seeds of decay of myriads of insects, leaves, plants, twigs, and branches. At every pace my head, neck, arms, or clothes were caught by a tough creeper, calamus thorn, coarse briar, or giant, thistle-like plant, scratching and rending whatever portion they hooked on. Insects also, of numberless species, lent their aid to increase my misery, especially the polished black ant, which affects the trumpet tree. As we marched under the leaves these ants contrived to drop on the person, and their bite was more vexatious than a wasp's or red ant's; the part bitten soon swelled largely, and became white and blistery. . . . These offensive sights and odours were met day after day, and each step taken was fraught with its own particular evil and annoyance; but with my present fading strength and drooping spirits they had become almost unbearable. . . . I had had no meat of any kind, of bird or beast, for nearly a month, subsisting entirely on bananas or plantains, which, however varied in their treatment by the cook, failed to satisfy the jaded stomach. My muscles had become thin and flabby, and were mere cords and sinews. Every limb was in a tremor while travelling, and the vitals seemed to groan in anguish for a small morsel of meat."

It is an established axiom with this journal that an ounce of practice is worth a pound of theory. Whoever has known the pangs of chronic dyspepsial and has experienced the sinking vacuum that stil, remains in the stomach even after what appears to be a whole cart-load of bread, or potatoes, or cabbage has been eaten, will sympathise profoundly with Stanley in his insatiable hunger for flesh. His language is like the tool of an engraver, and scores facts upon the memory so deeply that they can never be forgotten. "My muscles," says he, "had become thin and flabby, and were mere cords and sinews. Every limb was in a tremor while travelling, and the vitals seemed to groan in anguish for a small morsel of meat." The way to try the mettle and staying power of a horse is to put a heavy weight on his back in a rough hunting country, with a strong fox a mile or two ahead of the hounds. The horse which walks home fresh after a few stiff gallops and three or four hours following in such a country has proved his mettle. And the way to test a diet of meat or of vegetables is not to make the experiment upon a man in a City office, or a doctor trotting round for two or three hours daily in his brougham, but upon the literary man who produces two or three columns of strenuous "copy" daily, or upon the farm labourer who cuts down with his scythe an acre of meadow grass or two acres of wheat in a day; or, better still, upon a Stanley and his band who hew a pathway through the dense and terrible jungle in sweltering heat and miserable pains. The diet that stands this proves its unquestionable value; and the diet that, under such circumstances, brings a man nearer to death and the grave day by day is "weighed in the balances and found wanting."

The food question, when viewed in its true proportions, is seen to be of much greater importance than that of drugs. For whilst drugs are taken by a comparatively small number of the population, and that only occasionally, food is the everyday business of everybody in the world. Drugs could be entirely done without; and the change in current history would be so small as to be practically inappreciable. But if the food supply were to be stopped for a single month, the whole world would be depopulated. The questions at issue, therefore, between the flesh-eaters and the vegetarians are of the very first scientific rank.

There is no doubt whatever that flesh in some proportion is absolutely essential to the perfect health of man in most parts of the world. Universal experience is the proof of this; and universal experience is the very best evidence we can have on almost every subject on which man is required to decide. In religion, for example, an individual theorist is of absolutely no account whatever, when pitted against the common instincts and general sense of the whole human race. It is exactly the same with the food question. Science may modify and improve; and even mere abstract reasoning may sometimes suggest valuable ideas. But neither physical science nor formal logic can alter the course and constitution of human nature, or substitute mere plausible theories for natural and unchangeable facts. Vegetarianism, by way of an occasional change, and in strict subordination to the sound general practice of flesh-eating, may undoubtedly be both advantageous and pleasant. Here and there, too, there may be found a man or woman who can retain health, and even improve in strength on an exclusively vegetable diet. But that any person should have so little sense as to suppose that because vegetarianism has been of service to him during a single year, it is thereby proved that it would be of service to the whole world for all future time, is astonishing indeed. As well might a near-sighted man insist that all the world shall wear spectacles, because he can see three miles off with suitable glasses, but not three yards off without them.

A hobby to most men is like the wild steed to which Mazeppa was bound. Once they mount on its back they are entirely at its mercy. Many a man's intellect has been destroyed by being given up to a partial and limited experience. The very noblest ideals have in them the elements of danger. Taking religion again as our example, what moral, intellectual, and even physical ruin has not followed in its train when it has degenerated into unreasoning fanaticism! The same thing, though of course in a much less extensive degree, may be said of total abstinence. And even vegetarianism, although many people regard it as the most harmless of all "fads," is yet capable of doing vast injury to large numbers of people whose brains are feebler than their imitative impulses. Stanley's experiences may give pause to those who are labouring with apostolic zeal to turn the world upside down in favour of their peculiar modification in human diet. They never can succeed in converting the world to their way of thinking and acting; but they may succeed in persuading a considerable number of ill-instructed persons to enter upon a course which may undermine the health and even destroy the life. We are not among those who stand by the rule that nobody shall venture to express an opinion on any subject unless he has had a special training; but we do affirm that every man should understand that a certain responsibility attaches to him who teaches principles opposed to what are universally accepted, and that he must be prepared to accept his responsibility to the full. Every man has a right to teach who has the capacity and the knowledge; but he must remember that his doctrines, like Noah's dove, will sooner or later return to the ark from which they set out. Let vegetarians extol the virtues of fruit and vegetables as much as they please, but let them remember that bronze money can never possess the same value as gold, or be a practically adequate substitute for it.

